# Coronary flow and perfusion pressure

**DOI:** 10.4103/0253-7613.48877

**Published:** 2009-02

**Authors:** Pradeep Kumar, Manish Goyal

**Affiliations:** Department of Physiology, CSM Medical University, Lucknow, India; 1 All India Institute of Medical Sciences, New Delhi, India. E-mail: jenypradeep2002@yahoo.com

Sir

I read carefully an article published in the Indian Journal of Pharmacology, Apr 2008 vol. 40 Issue 2, entitled ‘Effects of low level lead exposure on blood pressure and function of the rat isolated heart’.[1]

The author has stated that the sub chronic exposure of lead does not show significant change in coronary flow in isolated perfused rat heart. It is well established that when oxygen consumption is kept constant, coronary blood flow is constant, independent of the coronary arterial pressure variations. When the pressure is kept constant, the flow varies linearly, with variations in oxygen consumption.[2] Variations in coronary arterial flow are directly coupled to the contraction-related intramural blood volume variations.[3] The interaction between flow variations and contraction is referred to as the intramyocardial pump.[4] Various metabolic substances, including vasodilators, produced locally at increased heart rate, can affect the perfusion pressure.[5] Therefore, to asses the coronary flow, tachycardia and positive inotropy is observed in this study. It is essential to keep constant perfusion pressure in isolated heart model, which was not done by the author. Hence, the author's statement is not justified.
